# Salmonella enteritidis Phlegmon in an Elderly Female: A Case Report

**DOI:** 10.7759/cureus.27545

**Published:** 2022-08-01

**Authors:** Zurabi Zaalishvili, Tamar Didbaridze, Nino Gogokhia, Besik Asanidze, Lali Akhmeteli, Liana Saginashvili, Giorgi Maziashvili

**Affiliations:** 1 Faculty of Medicine, Tbilisi State Medical University, Tbilisi, GEO; 2 Clinical Microbiology, Tbilisi State Medical University the First University Clinic, Tbilisi, GEO; 3 Microbiology Department, Tbilisi State Medical University, Tbilisi, GEO; 4 Laboratory Medicine, Tbilisi State Medical University the First University Clinic, Tbilisi, GEO; 5 General Surgery, Tbilisi State Medical University the First University Clinic, Tbilisi, GEO; 6 Surgery, Tbilisi State Medical University the First University Clinic, Tbilisi, GEO

**Keywords:** atypical infection, bacteremia, abscess, salmonella, phlegmon

## Abstract

*Salmonella enteritidis* is an individual serotype of *S. enterica* which can cause gastroenteritis in humans. In the case of a mild primary infection, bacteremia and phlegmon, as well as other types of extraintestinal Salmonella infection, may go undiagnosed.

A 64-year-old female presents with a one-week history of fatigue, fever, and low back pain. She recently noticed a progressively growing mass in her lower back, along with swelling and redness of the surrounding skin. The patient is a nursing home resident who has been immobilized since a fall one month before the presentation. The bacterial culture of discharge from the infected area was found to be positive for *S. enteritidis*, and the diagnosis of the torso phlegmon was made. The patient underwent surgical removal of the phlegmon and clinically improved after post-operative treatment.

After evaluating geographic location, time of the year, and host factors such as relative immobility, extremes of age, and immunosuppressive conditions, *S. enteritidis* should be considered in a differential diagnosis of torso phlegmon.

## Introduction

*Salmonella enterica* is a Gram-negative, facultative anaerobic rod. It spreads via the fecal-oral route and can be passed on through contaminated food and drink, direct animal contact, and, in rare cases, from person to person [1]. More than 2600 *S. enterica* serovars have been identified since the emergence of the disease in the 19th century, with many of these serovars capable of causing diseases in both humans and animals [2]. Enteric fever is caused by the human limited serovar Typhi and the closely related serovar Paratyphi-A, whereas non-typhoidal salmonellosis is caused by the generalist serovars Typhimurium and Enteritidis [3]. Mousselli et al. described epidural phlegmon and iliopsoas abscess cases where *S. enterica* was identified as the causative organism [4]. However, there are no documented reports of *S. enteritidis* causing phlegmon. Here, we report a case of a 64-year-old female who developed *S. enteritidis* phlegmon on the torso.

## Case presentation

A 64-year-old woman was hospitalized at the Tbilisi State Medical University (TSMU) The First University Clinic due to fatigue, fever, and low back pain on May 14, 2022. Her symptoms started one week ago. She noted a progressively increasing mass in her lower back with concomitant swelling and redness of the surrounding skin. She is a nursing home resident and has been bedbound since a fall one month before the presentation. Her past medical history is negative for recent gastrointestinal infections, diabetes, or immunocompromised states. However, it is significant for osteoarthritis treated with as-needed ibuprofen and hypertension diagnosed seven years ago and treated with captopril 25 mg since then.

On admission, her vital signs were significant for a temperature of 38.0 ℃ (100.4 ℉). The physical examination revealed a fluctuating mass (20 cm × 15 cm) with surrounding erythema and edema in the left lower back along the scapular line. A presumptive diagnosis of torso phlegmon was made and the following studies were ordered: complete blood count (CBC), basic metabolic panel (BMP), coagulation panel, C-reactive protein (CRP), chest X-ray (CXR), spinal MRI - T2 (FRFSE), T1 (FSE), STIR, viral hepatitis panel, HIV antibody test, VDRL, blood culture, and bacterial culture of pus from the mass.

Written informed consent was obtained from the patient for the publication of images or data included in this article.

Laboratory tests revealed abnormalities in the CBC and CRP, as shown in Table 1. White blood cells (26.07 × 10^9^/L), neutrophils (86%), and CRP (159.6 mg/L) were all elevated. The hemoglobin level (10.3 g/dL), the percentage of lymphocytes (11.6%), and the hematocrit (32.5%) were all lower than normal.

**Table 1:** Abnormal laboratory values

	Patient’s lab values	Normal lab value
White blood cell	26.07 × 10^9^/L	4.5 × 10^9^/L to 11 × 10^9^/L
Lymphocytes	11,6%	20–40%
Neutrophils	86%	40–60%
Hemoglobin	10.3 g/dL	12–16 g/dL
Hematocrit	32.5%	36–46%
C-reactive protein	159.6 mg/L	<10 mg/L

**Figure 1:**
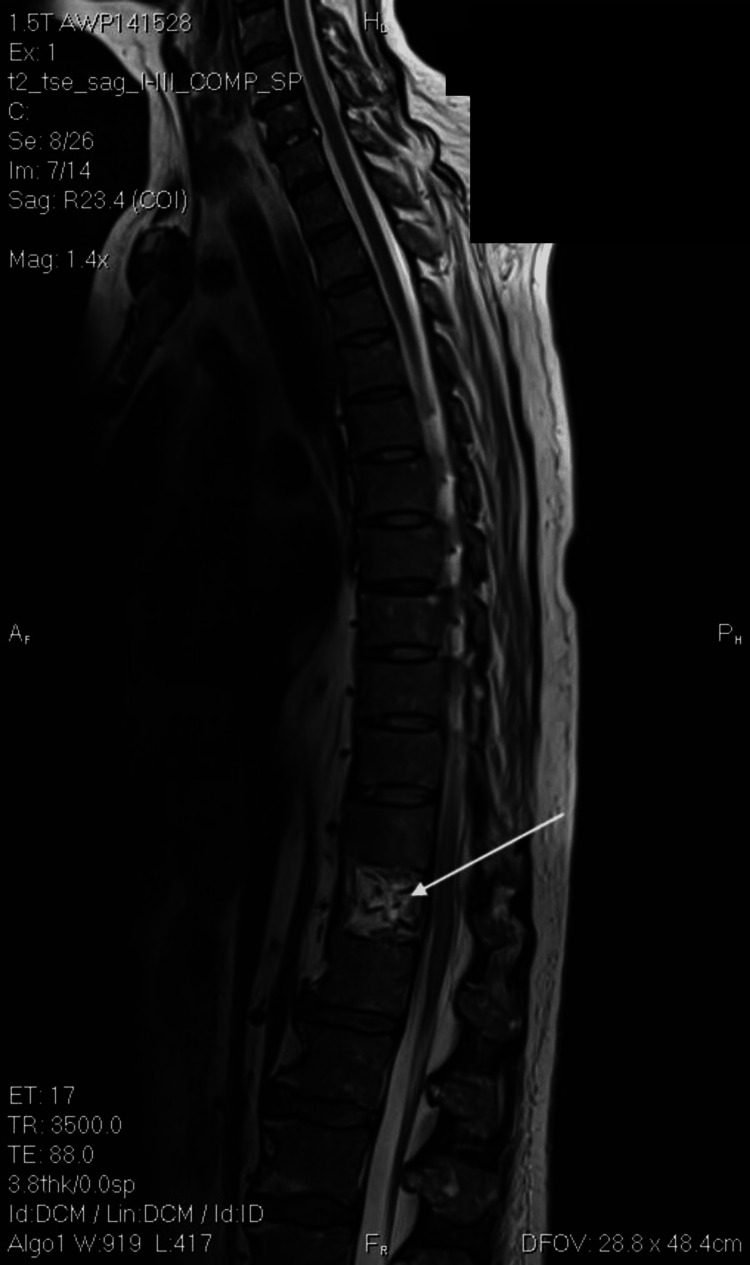
Magnetic resonance imaging of the spine in sagittal view. The white arrow indicates compression fracture.

**Figure 2:**
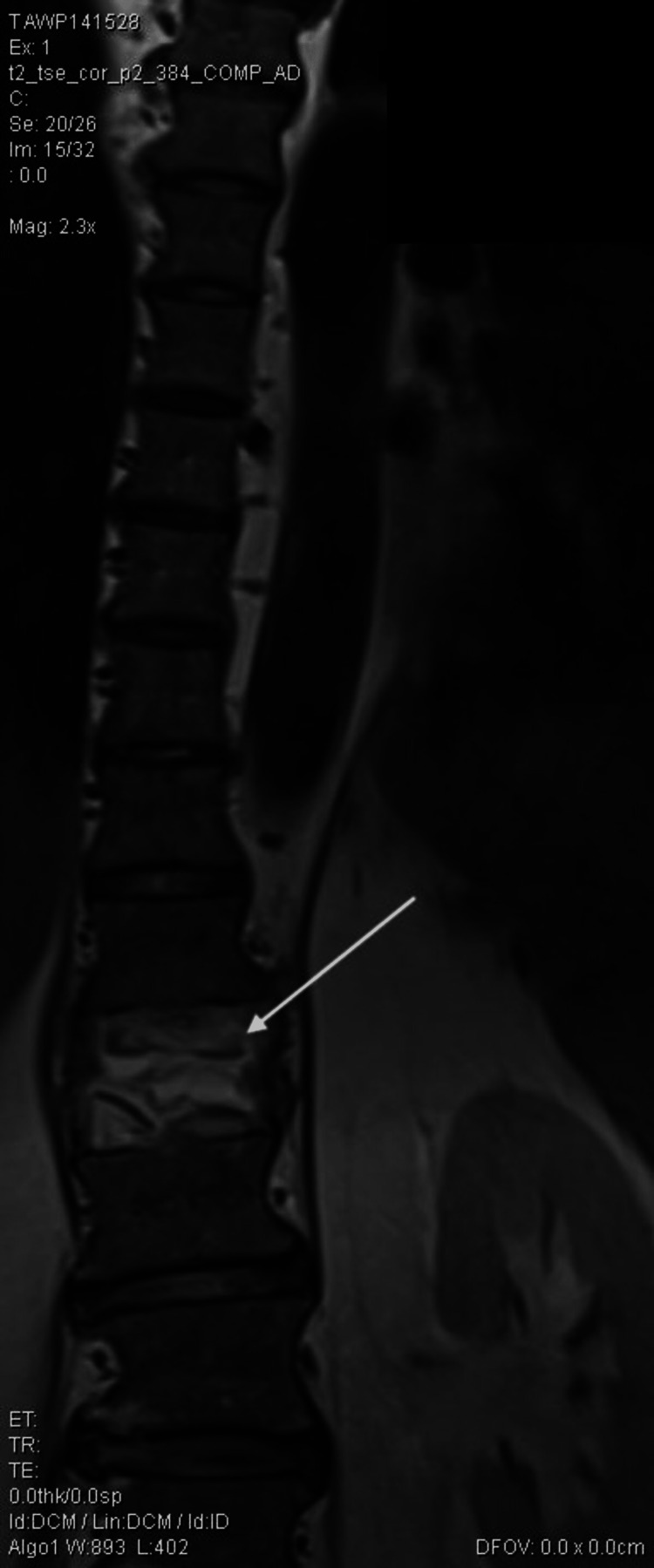
Magnetic resonance imaging of the spine in coronal view. The white arrow indicates compression fracture.

Spinal magnetic resonance imaging (MRI) findings were nonspecific except for the previous T12 compression fracture, as seen in Figures 1-2. The bacteriology laboratory of the clinic identified Salmonella spp. (10^8^ CFU/ml) using the Analytical Profile Index (API) manual identification system (BioMérieux, Marcy-l'Étoile, France). Identification of the pathogen from the pus confirmed the diagnosis of torso phlegmon. The isolate was then sent to the reference laboratory for a serology study, which identified Salmonella enteritidis. The blood culture was negative. The European Committee on Antimicrobial Susceptibility Testing (EUCAST) standard version 12 was used to assess antibiotic susceptibility against the following antibiotics: ceftriaxone, cefepime, ciprofloxacin, levofloxacin, piperacillin-tazobactam, amikacin, imipenem, meropenem. As seen in Table 2, the identified pathogen was sensitive to every tested antibiotic.

**Table 2:** Antibiotic susceptibility testing using the disc diffusion method (EUCAST Guidelines) EUCAST: European Committee on Antimicrobial Susceptibility Testing

Antibiotics (generic)	Interpretation
Ceftriaxone	SENSITIVE
Ciprofloxacin	SENSITIVE
Levofloxacin	SENSITIVE
Moxifloxacin	SENSITIVE
Amikacin	SENSITIVE
Piperacillin-Tazobactam	SENSITIVE
Imipenem	SENSITIVE
Meropenem	SENSITIVE
Cotrimoxazole	SENSITIVE
Cefepime	SENSITIVE

Other studies mentioned above did not reveal any significant findings. A general surgeon and a neurosurgeon were consulted. On May 15, 2022, the patient underwent surgical removal of phlegmon under general anesthesia. The procedure was performed without complications. The patient was transferred into the postoperative care unit with the following treatment regimen: ceftriaxone 1.0 × 2; metronidazole 500 mg/100 mL; enoxaparin 40 mg/0.4 mL; pantoprazole 40 mg × 1; diphenhydramine 10 mg/mL; captopril 25 mg; and metoclopramide 2 mL.

The patient improved significantly during the next five days in the hospital under the regular supervision of a general surgeon and an infectious disease specialist. The postoperative wound was cleaned daily with betadine solution. The patient was discharged on May 20, 2022, after proper counseling and education to prevent pressure ulcers and further torso infections.

## Discussion

Salmonellae belong to Gram-negative and facultatively anaerobic Enterobacteriaceae. The genus Salmonella is divided into two species: *S. enterica* and *S. bongori* [5]. *S. enterica* is further subdivided into six subspecies. Most clinically important salmonellae are formally classified into a single subspecies, *S. enterica*, subspecies enterica [5]. *S. choleraesuis*, *S. typhi*, *S. typhimurium*, and *S. enteritidis* are now identified as individual serotypes of this single subspecies [6].

Antimicrobial resistance patterns in nontyphoidal salmonellae vary significantly across the globe. Resistance to fluoroquinolones or third-generation cephalosporins is not widespread in the United States or Europe, but it warrants close monitoring. Salmonellae with extended-spectrum beta-lactamase (ESBL) genes are emerging in some areas [5].

Salmonellae can cause a variety of clinical infections, such as gastroenteritis, bacteremia, osteomyelitis, abscesses, and, rarely, phlegmon. Bacteremia and phlegmon, along with other types of extraintestinal Salmonella infection, may go unnoticed in the case of a mild primary infection [7]. Salmonella serotype, geographic location, time of year, and host factors such as relative immobility, extremes of age, and immunosuppressive conditions all influence the occurrence of bacteremia [7,8]. 

Our patient, who is a resident of a nursing home facility with poor sanitary conditions, developed *S. enteritidis* phlegmon on her lower back after being bedbound for one month. She had a stage 2 pressure ulcer on her back before the presentation, which could have been the source of infection. The abscess could be considered in a differential diagnosis; however, since the lesion was spreading along the tissue, this diagnosis has been ruled out. She reported no history of recent gastrointestinal infection, diabetes mellitus, or steroid administration. Although Salmonella phlegmon is relatively rare compared to other extraintestinal Salmonella infections, this case confirms how the patient’s comorbidities and poor sanitary conditions may predispose them to atypical infections. Additionally, physicians should keep a high index of suspicion for nontyphoidal Salmonella abscess/phlegmon when dealing with elderly immobile patients with infected subcutaneous masses.

## Conclusions

*S. enteritidis *is most commonly associated with gastroenteritis. However, it is imperative to include this infection in the differential diagnosis for phlegmon in bedbound patients with a poor hygienic environment. Further spread of the infection can be effectively prevented with quick recognition, diagnosis, and treatment. We believe that educating the patients plays an equally crucial role in preventing such atypical infections in the future. 
